# Cyanobactericidal Effect of *Streptomyces* sp. HJC-D1 on *Microcystis auruginosa*

**DOI:** 10.1371/journal.pone.0057654

**Published:** 2013-02-27

**Authors:** Yun Kong, Xiangyang Xu, Liang Zhu

**Affiliations:** 1 Department of Environmental Engineering, Zhejiang University, Hangzhou, China; 2 ZJU-UWA Joint Centre in Integrated Water Management and Protection, Hangzhou, China; Belgian Nuclear Research Centre SCK/CEN, Belgium

## Abstract

An isolated strain *Streptomyces* sp. HJC-D1 was applied to inhibit the growth of cyanobacterium *Microcystis aeruginosa* FACHB-905. The effect of *Streptomyces* sp. HJC-D1 culture broth on the cell integrity and physiological characteristics of *M. aeruginosa* FACHB-905 was investigated using the ﬂow cytometry (FCM), enzyme activity and transmission electron microscopy (TEM) methods. Results showed that the growth of *M. aeruginosa* FACHB-905 was significantly inhibited, and the percentage of live cells depended on the culture broth concentration and exposure time. The activities of antioxidant enzymes including superoxide dismutase (SOD), peroxidase (POD) and catalase (CAT) increased with exposure concentration and exposure time, and the significant increase of reactive oxygen species (ROS) led to the disruption of the subcellular structure of *M. aeruginosa* FACHB-905, and caused the increase of malondialdehyde (MDA). Furthermore, TEM observation suggested the presence of three stages (cell breakage, organelle release and cell death) for the cyanobactericidal process of *Streptomyces* sp. HJC-D1. Therefore, *Streptomyces* sp. HJC-D1 not only affected antioxidant enzyme activities and ROS level, but also destroyed the subcellular structure of *M. aeruginosa* FACHB-905, demonstrating excellent cyanobactericidal properties.

## Introduction

Up to now, the increasingly frequent outbreak of cyanobacterial blooms in lakes, reservoirs and rivers has drawn great attention in China [1]. Environmental and health problems caused by cyanobacterial blooms have been documented in many regions, and many eutrophication control methods such as chemical algaecides, oxidants, allelochemicals and cyanobactericidal microorganisms, have been applied for cyanobacteria and algae suppression [2], [3]. In the recent years, cyanobactericidal microbial technology has been regarded as a novel and safe method for eutrophic water remediation because of its environmentally friendly characteristics and efficiency.

Previous studies indicated that the inhibition of harmful algal or cyanobacterial growth might be the result of extracellular secretions from microorganisms [4], [5], [6]. These cyanobactericidal microorganisms including *Bacillus*, *Dietzia*, *Janibacter*, *Micrococcus* [5], *Streptomyces* sp. [4], [6], fungi [7], *Pseudoalteromonas* [8] and so on. The biodegradation mechanism of cyanobactericidal microorganism is speculated through direct or indirect attack [7], [8], such as the allelochemical inhibition on the growth of cyanobacteria [9], [10]. Several studies have shown that excess production of reactive oxygen species (ROS) in cyanobacteria occurs under environmental stresses, and the growth of cyanobacteria is inhibited [9], [11], [12], [13]. It was reported that exposure to atrazine increased the level of the malondialdehyde level (MDA) in *Chlorella vulgaris*, and the antioxidant enzyme activities including superoxide dismutase (SOD), catalase (CAT) and peroxidase (POD) were also increased markedly in the presence of atrazine [14]. At the same time, the oxidative damage described as lipid peroxidation might be one of the causes for the allelopathic effect of allelochemical gramine and pyrogallic acid on *M. aeruginosa* [9], [15]. However, it is difficult to distinguish different statuses of cyanobacterial cells by traditional methods such as cyanobacterial cell number or Chlorophyll *a* (Chl *a*) concentration under limited conditions, and no cyanobactericidal microorganism has been reported to induce antioxidant enzyme systems. Therefore, research on the enzymatic response of bloom cyanobacteria by cyanobactericidal microorganisms may be helpful to explain the cyanobactericidal mechanism and promote the application of cyanobactericidal microbial technology.

A bacterial strain named HJC-D1was isolated from an eutrophic pond in Hangzhou, China, and exhibited remarkable cyanobactericidal activity against *Microcystis aeruginosa*. It was identified as *Streptomyces* sp. by morphology and by 16S rRNA gene sequence analysis. In this study, the effect of *Streptomyces* sp. HJC-D1 culture broth on the cell integrity and physiological characteristics of *M.aeruginosa*, including the enzyme activities of SOD, CAT and POD, oxidative damage indicator of MDA and cellular redox status of ROS, were investigated through exposure tests using ﬂow cytometry (FCM), enzyme activity and transmission electron microscopy (TEM) technologies.

## Materials and Methods

### Cyanobactericidal Bacterium and Cyanobacterium Culturing

The strain *Streptomyces* sp. HJC-D1 with excellent cyanobactericidal activity characteristic used in this study was isolated from an eutrophication pond in Hangzhou, China. The culture of *Streptomyces* sp. HJC-D1 was maintained at 4°C in a Gause′s synthetic agar medium [16], and culture broth was prepared by incubating the seed culture at 28°C with a shaking speed of 150 rpm for 72 h. The *Streptomyces* sp. HJC-D1 fermentation broth was treated as follows before use: The mixture was centrifuged at 10,000×*g* for 10 min, and then filtered through a 0.22 µm cellulose acetate membrane to acquire a cell-free filtrate. The cell-free filtrate was subsequently inoculated into *M. aeruginosa* culture for cyanobactericidal activity tests.

*Microcystis aeruginosa* FACHB-905 was purchased from the Freshwater Algae Culture Collection of Institute of Hydrobiology (FACHB), Chinese Academy of Sciences (Wuhan, China). Before used as an inoculant, it was cultured for 7 d to reach the log phase under the following conditions: sterilized BG11 medium [17]; 2000 lux white light, light: dark = 14 h: 10 h; 25±1°C.

### Cyanobactericidal Activity Test of Isolated Bacterium on *M. aeruginosa* FACHB-905

The cyanobactericidal effects were studied by adding dilutions of *Streptomyces* sp. HJC-D1 culture broth (0, 1%, 3%, 5% and 10%, v/v) to a 500 mL sterilized conical beaker with 225 mL BG_11_ medium containing *M. aeruginosa* FACHB-905 cells at a Chl *a* concentration of 302.7±75.4 µg L^−1^, brought to a final volume of 250 mL by addition of Gause’s synthetic medium [16]. A negative control was made by adding 25 mL Gause′s medium into 225 mL cyanobacterial solution. All the samples and controls were incubated under the pre-set conditions described in Section “Cyanobactericidal bacterium and cyanobacterium culturing”. Each treatment was replicated three times and the arithmetical means (± SD) were obtained. Five mL of sample was filtered through the GF/F filter and then the chlorophyll was extracted using 10 mL of acetone (90%). The optical density of extracts were determined at 630, 645, 663 and 750 nm using a UV-2401 PC spectrophotometer (Shimadzu, Japan) with a 1 cm cell. The Chl *a* concentration of the *M. aeruginosa* culture was determined using the equations derived by reference [18].

### Flow Cytometric Analysis of Cyanobacterial Cells

Flow cytometric (FCM) analysis was employed for determining cell integrity of the tested cyanobacteria *M. aeruginosa*. The detailed procedures were described by Daly et al. [19], Chang et al. [20] and Prado et al. [21]. An FCM (Beckman Coulter Inc., Fullerton, CA, USA) equipped with an argon-ion excitation laser (488 nm) and forward (FS) and side (SS) light scatter detectors was employed. A standard green ﬂuorescence detector (FL1, 530 nm) was used to detect cells stained with SYTOX green nucleic acid stain (Invitrogen, USA) and ﬂuorescein diacetate (FDA) (Invitrogen, USA), and a red ﬂuorescence detector (FL4, 650 nm) was used to detect the auto-ﬂuorescence from chlorophyll in the cells. The ﬂow rate of cyanobacterial cells was controlled at 200 cells s^−1^ for each cytometric parameter investigated, and data were collected until the combined number of events recorded in the intact and ruptured regions reached 5000 or the analysis time reached 5 min. Data were collected using listmode files and statistically analysed using the EXPO32 ADC software (Beckman Coulter Inc.).

### Antioxidant Enzyme Activities Extraction and Analysis

Twenty-five millilitres of each culture was collected to extract enzymes following the method in Qian et al. [22]. Lipid peroxidation level was measured by MDA level according to Dogru et al. [23], while the measurement of SOD activity followed that of Trenzado et al. [24]. CAT and POD activity was measured according to Qian et al. [25]. The activity of each enzyme was expressed on a protein basis. The ROS level was determined by analyzing the ﬂuorescence intensity of 2′,7′-dichloroﬂuorescein (DCF) [22].

### Protein and Carbohydrate Analyses

Proteins were detected by the bicinchoninic acid (BCA) method, and carbohydrates were detected by the phenolesulphuric acid method [26].

### Transmission Electron Microscopy

Cyanobacterial samples were harvested by centrifugation at 10000×*g* for 5 min, the supernatant was discarded, and then cyanobacterial cells were fixed in 2.5% glutaraldehyde in Phosphate Buffer Solution (PBS) for 24 h at 4°C. After fixation, the samples were post-fixed in 1% buffered osmium tetroxide for 2 h, dehydrated using a graded ethanol and embedded in Epon-812. Ultrathin sections were stained in 2% uranyl acetate and lead citrate, and examined with a Hitachi H-600 transmission electron microscope [13], [27].

### Statistical Analysis

The results were expressed as mean ± SD. Statistical analysis was performed using Version 17.0 of SPSS for Windows (SPSS, Chicago, IL, USA). All data were analyzed using one-way ANOVA followed by the least significant difference test to evaluate cyanobacterial oxidative damage and antioxidant responses of the cyanobacterium. When the probability (*p*) was less than 0.05 or 0.01, the values were considered significantly different.

## Results

### Effect of *Streptomyces* sp. HJC-D1 on the Growth of *M. aeruginosa* FACHB-905

**Figure 1:**
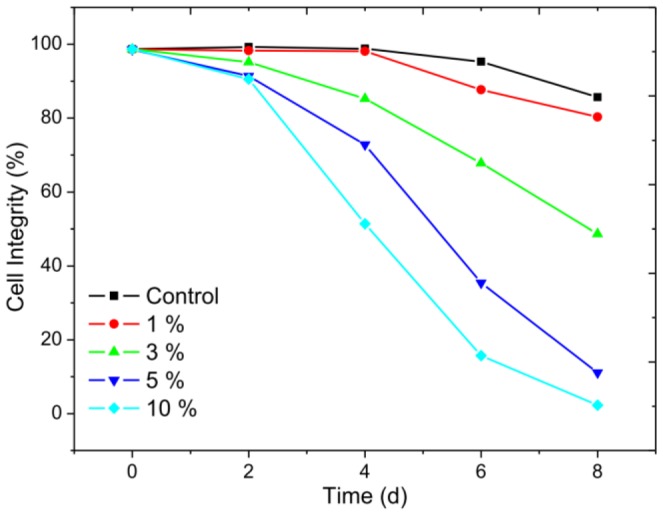
Cell viability for *M. aeruginosa* FACHB-905 with and without exposure to *Streptomyces* sp. HJC-D1 culture broth.

Flow cytometry was used to distinguish living cells from chlorotic cells resulting from exposure to different concentrations of *Streptomyces* sp. HJC-D1 culture broth. Fig. 1 shows the effect of *Streptomyces* sp. on the cell integrity of *M. aeruginosa* FACHB-905 after 8 d incubation. Before the addition of *Streptomyces* sp. HJC-D1, all the cells are integral. The growth of *M. aeruginosa* FACHB-905 cells were effectively inhibited by the culture broth with a concentration range of 3%–10% (v/v) after 4 d exposure. The growth of *M. aeruginosa* FACHB-905 was significantly inhibited and the percentage of live cells decreased with greater culture broth concentration and time of exposure, which were 51.4%, 15.7% and 2.3% for the 3%, 5% and 10% treatment group after 8 d exposure, respectively (shown in Fig. 1).

### Effect of *Streptomyces* sp. HJC-D1 on Cyanobacterial Protein and Carbohydrate Contents

**Figure 2:**
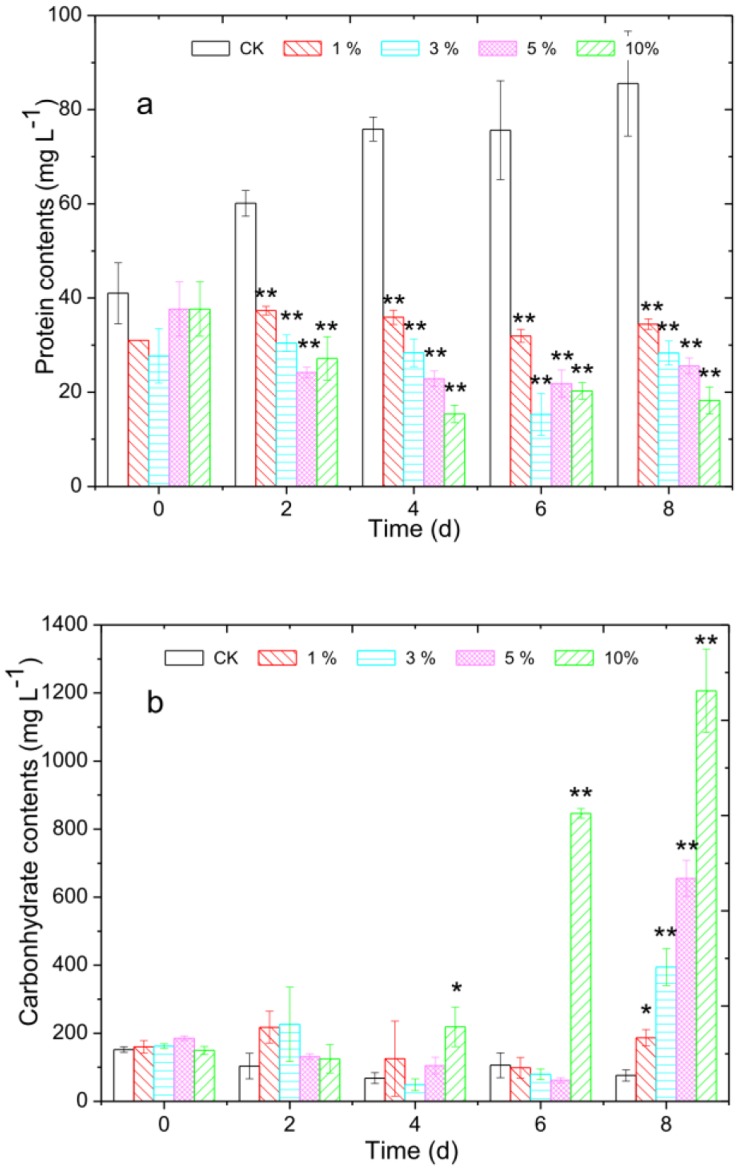
Effect of *Streptomyces* sp. HJC-D1 culture broth on cyanobacterial protein and carbohydrate contents. (a) protein contents; (b) carbohydrate contents. * represents a statistically significant difference of *p*<0.05 when compared to the control, ** represents a statistically significant difference of *p*<0.01.

The effects of *Streptomyces* sp. HJC-D1 on the protein and carbohydrate contents appear in Fig. 2. The protein contents of the cyanobacterial cells in the treatment groups that were exposed to culture broth (1%, 3%, 5% and 10%, v/v) were 35.90±1.53, 28.37±2.94, 22.87±1.62 and 15.38±1.84 mg L^−1^, which was 47.3%, 37.4%, 30.1% and 20.3% of the control group (75.90±2.55 mg L^−1^), respectively. For the 8 d incubation, protein content experienced almost no change compared to that of the 4 d incubation (Fig. 2a).

On the contrary, an obvious increase in carbohydrate contents was observed after *M. aeruginosa* FACHB-905 cell exposure to *Streptomyces* sp., although the increase was not obvious after 4 d incubation (Fig. 2b), and the carbohydrate content of cyanobacterial cells on day 8 was 76.12±16.49 mg L^−1^ for the control group and 86.80±23.65, 394.63±54.29, 655.60±53.16 and 1206.6±122.70 mg L^−1^ for the treatment groups, respectively.

### Effect of *Streptomyces* sp. HJC-D1 on Cyanobacterial MDA, SOD, POD, CAT Activities

**Figure 3:**
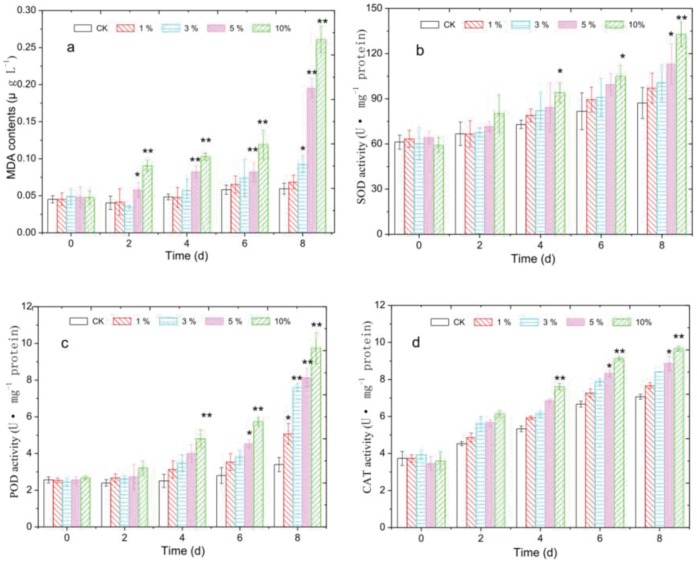
Effect of *Streptomyces* sp. HJC-D1 culture broth on MDA content and SOD, POD and CAT activities. (a) MDA contents; (b) SOD activities; (c) POD activities; (d) CAT activities. * represents a statistically significant difference of *p*<0.05 when compared to the control, ** represents a statistically significant difference of *p*<0.01.

Figure 3a shows that *Streptomyces* sp. HJC-D1 induced a significant increase in MDA contents in *M. aeruginosa* FACHB-905. Compared with the control group, the MDA content was 0.048±0.013, 0.057±0.015, 0.082±0.008 and 0.103±0.005 µg L^−1^ after 4 d co-culturing (0.048±0.004 µg L^−1^ for the control) and 0.068±0.015, 0.082±0.012, 0.191±0.060 and 0.272±0.018 µg L^−1^ after 8 d incubation (0.052±0.007 µg L^−1^ for the control) for the treatment groups (1%, 3%, 5% and 10%), respectively. The ratio of MDA of the treatment versus the control group increased from 1.00 (1%), 1.19 (3%), 1.71 (5%) and 2.15 (10%) to 1.31, 1.58, 3.67 and 5.23, respectively.

Cellular enzymatic activities including SOD, POD, and CAT were determined to investigate the cellular defense response induced by *Streptomyces* sp. HJC-D1 stress. As shown in Fig. 3b, SOD activity showed a significant increase with the increase in culture broth concentration. The activity values were 1.13, 1.19, 1.22 and 1.31 times (*p*<0.05) greater than the control when cyanobacterial cells were treated with *Streptomyces* sp. HJC-D1 of 1%, 3%, 5% and 10% after 4 d respectively. As exposure time increased, the SOD activity for the 10% treatment group was enhanced from 97.3±4.8 U mg^−1^ protein on day 4 to 132.4±7.1 U mg^−1^ protein on day 8 versus the control group with the maximum increase of 140.6% in response to *Streptomyces* sp. HJC-D1. It showed that the effect of both POD and CAT activities were similar to that of the SOD activity: the activities increased with higher culture broth concentration. After exposure to 1%–10% of *Streptomyces* sp. HJC-D1 for 4 d and 8 d, POD activity increased from 1.45 to 2.18 times and 1.61 to 2.85 times (Fig. 3c), while the CAT activity increased from 1.11 to 1.62 times and 1.08 to 1.39 times, respectively (Fig. 3d). Prolonged exposure time had an obvious inﬂuence on the CAT activity, however, the variation tendency for CAT activity was not obvious compared to POD activity.

### Effect of *Streptomyces* sp. HJC-D1 on ROS Level

**Figure 4:**
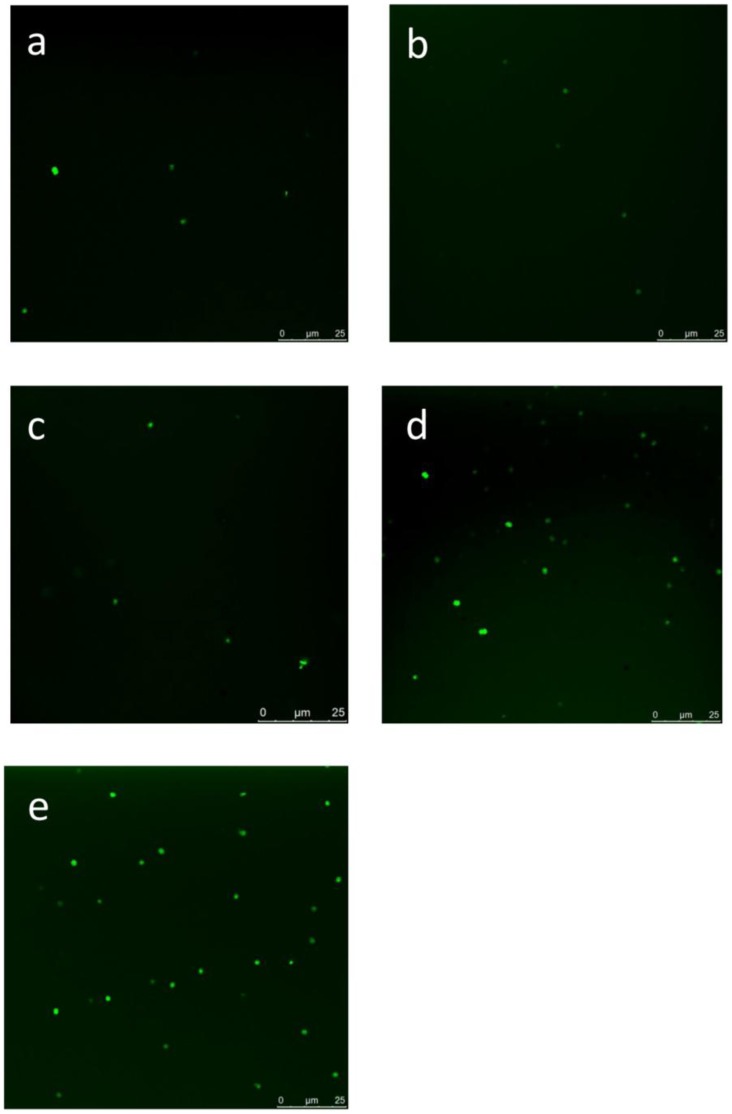
The effect of *Streptomyces* sp. HJC-D1 on the formation of intracellular ROS in *M. aeruginosa* FACHB-905. The intracellular ROS were detected with laser confocal microscope after DCFH-DA treatment. (a) The control of *M. aeruginosa* FACHB-905; (b), (c), (d) and (e) was the treatment of *M. aeruginosa* FACHB-905 with different concentration of 1%, 3%, 5% and 10% (v/v), respectively.

To investigate whether the cells of *M. aeruginosa* FACHB-905 were in oxidative stress, the growth of *M. aeruginosa* FACHB-905 inhibited by *Streptomyces* sp. HJC-D1 was examined in terms of the ROS level. Weak ﬂuorescence of DCF was present in the control group (Fig. 4a), and ﬂuorescence intensity was significantly increased after exposure of fresh cyanobacterium to *Streptomyces* sp. HJC-D1 after 8 d (Fig. 4b, c, d and e). The increase in ROS level was positively correlated with *Streptomyces* sp. HJC-D1 concentration.

### Effect of *Streptomyces* sp. HJC-D1 on Subcellular Structure

**Figure 5:**
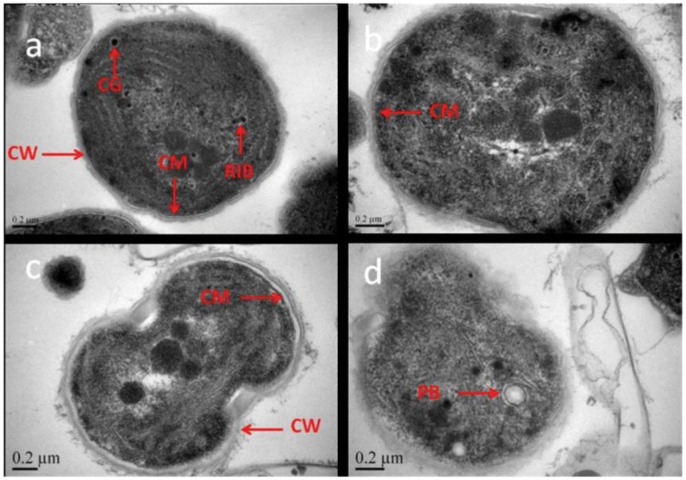
Biological degradation process of *M. aeruginosa* FACHB-905 by *Streptomyces* sp. HJC-D1: (a) normal cell; (b) cell walls becoming detached; (c) release of cellular contents; (d) broken cell. CW, cell wall; CM, cell membrane; PB, polyphosphates bodies; CG, cyanophycin granules; RIB, ribosomes.

TEM analysis was used for evaluating the effect of *Streptomyces* sp. HJC-D1 on the surface morphology of the cyanobacterial *M. aeruginosa* FACHB-905. The ultrastructure of *M. aeruginosa* FACHB-905 was compared between control cells and those exposed to *Streptomyces* sp. HJC-D1 culture broth (5%, v/v) for 4 d (shown in Fig. 5a), and results showed that the entire surface of *M. aeruginosa* FACHB-905 cell was enclosed by cell wall. The cell membrane was close to the cell wall, and the photosynthetic lamellae was in uniformity. However, the surface of *M. aeruginosa* FACHB-905 cells changed distinctly after the exposure to *Streptomyces* sp. (shown in Fig. 5b–d). Compared to the control cell, the cell membrane of *M. aeruginosa* was detached from the cell wall (shown in Fig. 5b), and the photosynthetic lamellae became nonuniform. Soon afterwards, the cell wall of *M. aeruginosa* began to break down and organelles such as polyphosphates bodies, cyanophycin granules, ribosomes and thylakoids were released from the cell (shown in Fig. 5c). As one of the most important organelles, the photosynthetic lamellae structure was also disrupted with the alteration and impairment of the thylakoids. Finally, the cellular structure entirely disappeared, and the cell membrane of *M. aeruginosa* was degraded completely while much of the contents leaked out (shown in Fig. 5d).

## Discussion

As a rapid and sensitive technique to distinguish different statuses of cyanobacterial cells, FCM can measure cell number and various physiological characteristics of individual cells by using appropriate fluorescent probes. FCM has been used to distinguish subpopulations of cells exposed to tibetan hulless barley or herbicide [19], [20], [21], [28], [29]. In the present study, the analysis by means of ﬂow cytometry showed that the different concentration of culture broth in the culture medium inhibited the growth of *M. aeruginosa* FACHB-905, and cyanobacterium cultures with culture broth concentrations of greater than or equal to 5% (v/v) showed a good inhibition performance compared to the control group. Similar to our results, Hua et al. [4] found that *Streptomyces* strain NT0401 caused a significant reduction in live *M. aeruginosa* cells at 5% (v/v) treatment level. The cyanobactericidal activity of microorganisms towards many kinds of cyanobacteria have also been published in other recent studies [5], [7], [30], and the effective removal efficiencies of Chl *a* were reported as approximately 50%–98% at a suitable bacterial density for more than 6 d [30], [31]. However, cell viability was restored after treatment for a period of time [10].

In spite of a heightened interest into the physiology and metabolism of cyanobactericidal bacteria and the improved availability of genome data, knowledge on the underlying molecular mechanisms, i.e., in respect to variations in antioxidant enzyme activities, remains rather limited. Hong et al. [32] have pointed out that membrane lipid peroxidation was a vital sign of cellular damage, and most antioxidant enzyme activities increased under stress conditions. Cell membranes made of unsaturated phospholipids often experienced membrane lipid peroxidation in adverse situations, and were vulnerable to oxygen attack resulting in MDA accumulation [14], [27], [32]. In the present study, *Streptomyces* sp. HJC-D1 could improve the MDA levels, and MDA content increased with increasing culture broth (Fig. 3a), indicating that *Streptomyces* sp. HJC-D1 induced membrane lipid peroxidation and caused damage to cell membranes; this phenomenon was also found by Hong et al. [9], [33] and Qian et al. [14], [34]. It was obvious that the activities of antioxidant enzymes such as SOD, POD and CAT also indicated a significant increase in exposure to *Streptomyces* sp. HJC-D1 (Fig. 3b, c and d). Previous research suggested that the consistent increase in SOD, CAT, and POD activities in algae meant these antioxidant enzyme activities might be an important site of action of atrazine or glufosinate on *C. vulgaris* [14], [34]; the same variation in antioxidant enzyme activities was also found when the cyanobacteria or algae were exposed to allelochemicals [15], [27], [33].

It is well known that the superoxide anion radical, hydrogen peroxide, and hydroxyl radicals that belong to ROS are produced during membrane-linked electron transfer localized in mitochondria, chloroplasts and peroxisomes [10], [12], [35]. In addition, the oxidative damage was caused either directly or indirectly by triggering an increased level of ROS [12], and one of the responses of cyanobacteria and green algae to cyanobactericidal stress is the excess production of ROS [11]. Therefore, the acute increase in ROS level in the study showed that the *M. aeruginosa* FACHB-905 cells were in serious oxidative stress (Fig. 4). Futhermore, the increased ROS levels resulted in oxidative damage on membrane lipids, nucleic acids and protein (Fig. 4). This phenomenon has been observed in other interactions between cyanobacteria and allelochemicals. A previous study reported that the ROS level of the cyanobacterium *M. aeruginosa* after treatment rose remarkably to 1.91 times that of the controls at high ethyl 2-methyl acetoacetate (EMA) concentration of 4 mg L^−1^ for 2 h, with an increase in the ROS level occurring after 24 h [32]. It could be found that the gramine caused an obvious increase in the ROS level of *M. aeruginosa* cells [9]. The results were consistent with the previous studies [32] in the sense that both an increased permeability of membranes as well as the damage to the cellular membrane structure were present.

The growth of cyanobacterial, algal, and diatomic cells is generally associated with the production of Chl *a*, protein and carbohydrate [36] and levels of cellular protein and carbohydrate are two basic indicators to reﬂect the physiological state of algae cells [10], [21]. The increase of cellular protein content for the control group in the experiment indicated that new protein was synthesized; however, with extended *Streptomyces* sp. HJC-D1 treatment time, the contents of protein for the treatment groups began to decrease (Fig. 2a). This finding implied that the cells were not adapted to the external environmental stress, and the result was similar to the use of allelochemicals such as EMA [10]. However, the changes to carbohydrate contents were different from that of protein. The carbohydrate contents increased significantly with increasing culture broth concentrations (Fig. 2b), and the major reason for the biodegradation of cyanobacterium cells was *Streptomyces* sp. HJC-D1, which was consistent with the results in section 3.5. It was also found that carbohydrate contents increased significantly when *M. aeruginosa* was exposed to EMA for 3 d [10], thus supporting observations in the current study.

Generally, the cyanobacterial organic matter is comprised of proteins, neutral and charged polysaccharides, nucleic acids, lipids and small molecules, of which polysaccharides can comprise up to 80–90% of the total release [37]. It showed that cyanobacteria had more protein contents (41–69%) than diatoms (12–50%), while diatoms appeared to accumulate more lipids in the cells (5–43%) than cyanobacteria and green algae (2–30%) [38]. Henderson et al. [36] described that the ratio of protein to carbohydrate was 0.6 mg mg^−1^ at the stationary phase and 0.31 mg mg^−1^ at the exponential phase for *M. aeruginosa*, demonstrating that the amount of protein relative to carbohydrate decreased over time. Ultrastructural examination by TEM demonstrated that damage to *M. aeruginosa* FACHB-905 occurred. This result indicated that the cellular structure disappeared and caused the photosynthetic complex to disaggregate. The variation of the protein and carbohydrate contents implied that some organics were produced by the chlorotic *M. aeruginosa* FACHB-905 cells, and the increases of antioxidant enzyme activities and ROS implied that the destruction of the cell structure might be the imbalance of oxidative stress on antioxidant defense system in *M. aeruginosa* FACHB-905 caused by *Streptomyces* sp. HJC-D1 culture broth, which could be an indicator of the disintegration of the *M. aeruginosa* FACHB-905 cells, and thus in accordance with results obtained by ﬂow cytometry and TEM.

### Conclusion

An isolated strain named *Streptomyces* sp. HJC-D1 was used to inhibit the growth of *Microcystis aeruginosa*: its culture broth showed remarkable cyanobactericidal activity. The analytical method of flow cytometry was adopted to assess the physiological status of *M. aeruginosa* FACHB-905 cells during the cyanobacterial inhibiting process, and results showed that the density of live cells decreased markedly after exposure to the cyanobactericidal culture broth. TEM analysis showed that there were three stages (cell breakage, organelle release, and cell death) for the cyanobactericidal process of *Streptomyces* sp. HJC-D1, and the increase of SOD, POD and CAT activities and ROS level indicated that oxidant damage and the membrane integrity might be the cyanobactericidal mechanism of *Streptomyces* sp. HJC-D1 on *M. aeruginosa* FACHB-905. In view of these results, it’s concluded that *Streptomyces* sp. HJC-D1 not only affects antioxidant enzyme activities and ROS level, but also destroys the subcellular structure of *M. aeruginosa* FACHB-905, thus exhibiting excellent cyanobactericidal activity.
